# Pheochromocytoma multisystem crisis treated with emergency surgery: a case report and literature review

**DOI:** 10.1186/s13104-015-1738-z

**Published:** 2015-12-09

**Authors:** Katsura Kakoki, Yasuyoshi Miyata, Youhei Shida, Tomoaki Hakariya, Kosuke Takehara, Seiya Izumida, Motohiro Sekino, Naoe Kinoshita, Tsukasa Igawa, Junya Fukuoka, Hideki Sakai

**Affiliations:** Department of Urology, Nagasaki University, Graduate School of Biomedical Sciences, 1-7-1 Sakamoto, Nagasaki, 852-8501 Japan; Department of Cardiovascular Medicine, Nagasaki University Graduate School of Biomedical Sciences, Nagasaki, 852-8501 Japan; Division of Intensive Care, Nagasaki University Hospital, Nagasaki, 852-8501 Japan; Department of Pathology, Nagasaki University Hospital, Nagasaki, 852-8501 Japan; Department of Urology, Kurume University Hospital, Fukuoka, 830-0011 Japan

**Keywords:** Pheochromocytoma multisystem crisis, Treatment strategy, Outcome, Cell proliferation, Malignant pheochromocytoma

## Abstract

**Background:**

Pheochromocytoma is a neuroendocrine tumor that predominantly presents with hypertension, palpitations, and tachycardia due to excessive catecholamine excretion. Although pheochromocytoma multisystem crisis (PMC) is relatively rare, urologists and clinicians should focus on early diagnosis as delay in initiating the appropriate treatment can lead to mortality

**Case presentation:**

A 70-year-old man developed ileus after a few days of medication for hypertension. Computed tomography incidentally revealed a left adrenal mass. This finding together with his clinical course was compatible with pheochromocytoma. An α-blocker was administered immediately, and his blood pressure was well controlled. However, his general condition and laboratory data deteriorated rapidly, and the patient was diagnosed with PMC with lethal status. Thus, emergency adrenalectomy was performed without confirmation of catecholamine levels. From the resected specimen, his tumor was judged as pheochromocytoma. On immunohistochemical analysis, the proliferation index evaluated by Ki-67 staining was 9.7 %. This case report was approved by the Human Ethics Review Committee of the Nagasaki University Hospital.

**Conclusion:**

The present case of PMC was successfully treated with emergency surgery. The benign pheochromocytoma also presented with high cell proliferation potential, which may be a cause of the extreme aggressiveness of PMC.

## Background

Pheochromocytoma is a relatively rare neuroendocrine tumor that predominantly presents with proximal or sustained hypertension, palpitations, tachycardia, and sweating due to excessive catecholamine release. These symptoms are often severe, and some patients can enter ‘pheochromocytoma crisis’ [1, 2]. Patients judged to be in pheochromocytoma crisis require treatment with antihypertensive drugs and transfusion. The standard treatment for ‘non-crisis’ pheochromocytoma is generally preoperative preparation with an α-blocker and surgical resection [1].

Pheochromocytoma multisystem crisis (PMC) is a fatal condition characterized by multiple organ failure, severe blood pressure variability, high fever, and encephalopathy [2]. Importantly, PMC is not synonymous with malignant hypertension caused by the massive release of catecholamine’s from the pheochromocytoma. Indeed, several patients with PMC were reported to be normotensive or hypotensive [3, 4]. In addition, the symptoms and pathological conditions of PMC vary greatly. Abdominal pain, nausea, and dyspnea are common, while anemia, back pain, night sweat, and acidosis have also been reported [5]. Therefore, many patients with PMC are unrecognized at diagnosis [5]. The treatment strategy for patients with PMC requires particular attention, as delayed therapy can lead to severe sequelae or mortality.

Detailed information on the diagnosis and treatment of PMC is important for physicians. In addition, understanding the pathological characteristics of PMC is essential for developing diagnostic tools and treatment strategies. Unfortunately, there are few reports on the molecular biological findings or pathological characteristics in PMC patients [6]. Herein, we report a case of a patient with PMC who was successfully treated with emergency surgery. We also provide a literature review of the symptoms, data, treatments, and outcome of patients with PMC, as well as the relationship between cell proliferation and malignant potential in pheochromocytoma. The present case report presents new and important information for future studies on PMC. The authors obtained approval from the Human Ethics Review Committee of Nagasaki University Hospital for the publication of this report.

## Case presentation

**Fig. 1:**
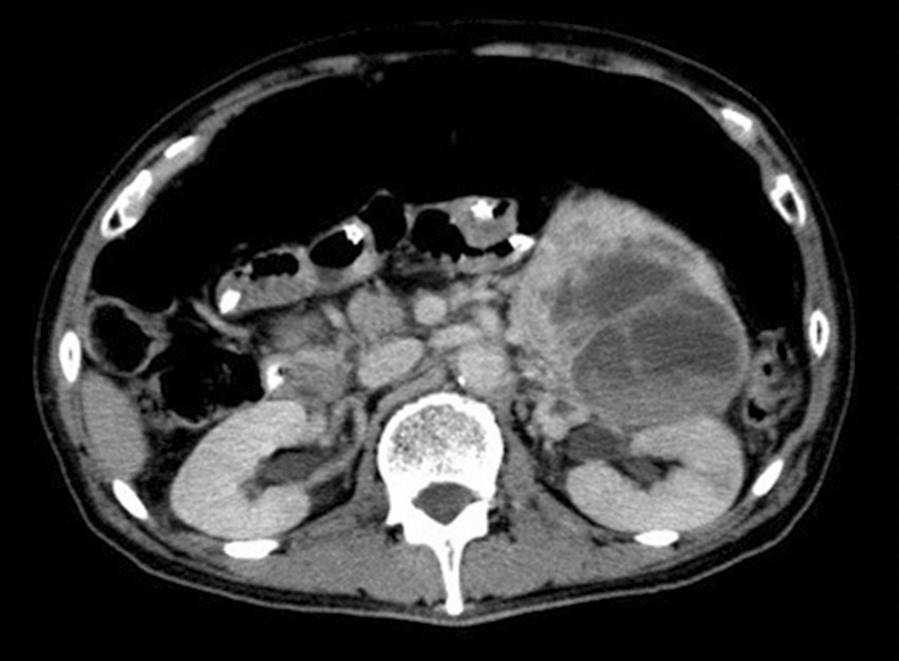
Abdominal computed tomography revealed a large left adrenal mass with heterogeneous enhancement and extended intensity compatible with ileus

A 70-year-old man was admitted to our hospital for ileus and uncontrollable high blood pressure. His systolic blood pressure exceeded 160 mmHg, and oral diltiazem and amilodipin were initiated 5 days before the onset of ileus. Computed tomography showed a left adrenal mass and ileus (Fig. 1). His consciousness level was low with incoherent speech. His body temperature was 37.4 °C, pulse rate was 126 bpm, blood pressure was 210/146 mmHg, and SpO^2^ was 98 % on room air. He appeared to be sweating profusely, with cold moist peripheries. Electrocardiogram showed sinus rhythm tachycardia and short PQ, high voltage, mitral P, and negative T on V4–6. Abdominal radiography showed large intestine expansion due to gas. Initial laboratory data showed low thrombocyte count (74,000/μL), normal white blood cell count (6000/μL), high levels of hemoglobin (16.3 g/dL), C-reactive protein (9.30 mg/dL), serum glucose (201 mg/dL), and blood urea nitrogen (48 mg/dL), and normal level of serum creatinine (1.05 mg/dL). Amino-terminal pro-brain natriuretic peptide was extremely high at 13,938.0 ng/mL.

We suspected pheochromocytoma on the basis of his computed tomography findings and clinical features. Doxazosin (2 mg/day orally), nicardipine (1.5 μg/kg/h i.v.), and landiolol (200 mg/day i.v.) were administered for blood pressure control. Because of the high fever, we suspected necrotizing enteritis due to ileus. Therefore, intravenous antibiotics were administered. Moreover, creatinine kinase and lactose dehydrogenase increased rapidly to 7066 and 609 IU/L, respectively. At that time, his body temperature was 40.1 °C. His respiratory state was aggravated after 3 days in the hospital, and oxygen was started. Laboratory tests showed that renal and liver functions were deteriorating. Five days after hospitalization, tracheal intubation was performed in the intensive care unit. Artificial ventilation and continuous hemodiafiltration were started.

The patient was diagnosed with PMC on the basis of the clinical course and radiological findings, without considering serum catecholamine levels. We performed emergency left adrenalectomy despite his critical condition, as he showed poor control and was refractory to medical treatment. Intraoperatively, the anesthesiologist used several types of antihypertensive drugs including doxazosin, landiolol hydrochloride, and diltiazem as necessary. Although temporary blood pressure was reduced to 80/46 mmHg after removal of the adrenal gland, the patient’s vital signs stabilized quickly after catecholamine infusion.

Postoperatively, the patient’s blood catecholamine levels intraoperatively were: epinephrine 10,351 (normal range ≤100 ng/mL), norepinephrine 27,654 ng/mL (normal range 100–450 ng/mL), and dopamine 209,500 ng/mL (normal range ≤20 ng/mL). The patient recovered rapidly postoperatively. One day postoperatively, his body temperature normalized. Furthermore, lactose dehydrogenase, creatinine kinase, liver function, respiratory status, and consciousness began to improve. He was weaned from ventilatory support at 2 days postoperatively, and from continuous hemodiafiltration and catecholamine administration at 3 days postoperatively. He was discharged 37 days postoperatively. Various imaging examinations including computed tomography and magnetic resonance imaging were performed during hospitalization to reconfirm the absence of metastatic mass. He has had no tumor recurrence or metastasis for 2 years.

**Fig. 2:**
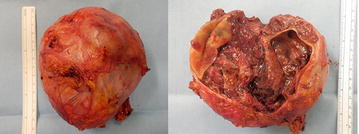
Resected specimen showing a tumor measuring 14 cm in diameter. There was hemorrhage and necrosis within the tumor

**Fig. 3:**
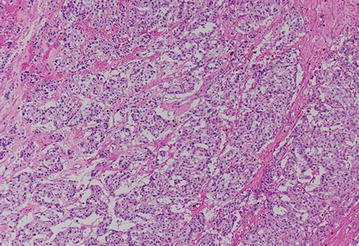
Hematoxylin-eosin staining (magnification: ×40)

**Fig. 4:**
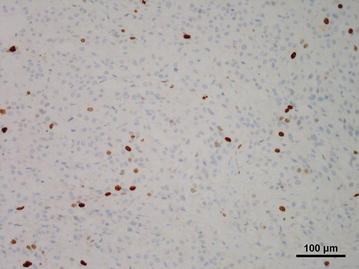
Ki-67 staining. The distribution of Ki-67-stained cells was nonspecific (magnification: ×200)

The resected tumor was 12 × 11 × 8 cm in size and weighed 530 g, with prominent central necrosis and internal bleeding (Fig. 2). Histologically, the tumor had a capsule and was comprised of basophilic granular cells with polygonal features (Fig. 3). On immunohistochemistry, the specimen stained positive for chromogranin A, NCAM, and S100. Therefore, the tumor was identified as a pheochromocytoma. The cell proliferation index measured using an anti-Ki-67 antibody was 9.7 % (Fig. 4).

## Discussion

The incidence of pheochromocytoma is approximately 0.1 % in the hypertensive population [7]. Pheochromocytoma typically arises from the adrenal medulla, although may develop in chromaffin cells in or around sympathetic ganglia [8]. The standard treatment for symptomatic pheochromocytoma is generally surgical resection. PMC is a more severe and potentially lethal condition of pheochromocytoma, and represents an endocrinological emergency [9]. Therefore, clinicians should have detailed information regarding the symptoms, diagnosis, and treatment of PMC, despite its rarity.

Several previous reports have stressed the importance of emergency treatment for PMC to avoid death [2, 6]. Indeed, all European cases of PMC without surgical resection (5/12, 41.7 %) died despite conservative treatment (Table 1). The results of physical examination and hormonal studies in previous PMC cases are shown in Table 1. Although extremely high blood pressure and fever are characteristics of PMC, two patients had no high-grade fever. Other signs and symptoms of PMC have been reported [5]. Therefore, accurate diagnosis on the basis of vital signs, symptoms, and physical examination can be difficult.

**Table 1:** Characteristics, physical conditions, treatments, and outcomes of patients with pheochromocytoma multisystem crisis

Sex	Age (years)	S/DBP (mmHg)	BT (°C)	Size (cm)	Ad	NAd	Dopa	Operation (days)^b^	Outcome	Year [Ref]
					(Times of upper limit)					
F	53	210/110	40	6.3	101.1	103.5	–	Not performed	Died	1988 [2]
F	62	285/140	40.5	10	96.0	14.8	–	Emergency	Survived	1988 [22]
F	50	200/90	40	7	43.3	23.4	–	7	Survived	1988 [2]
F	65	120/80	39.5	4	–	–	–	>21	Survived	1993 [10]
M	31	164/40	41.0	–	10^a^	6.7^a^	1.3^a^	36	Survived	1997 [11]
F	50	160/100	41.5	–	–	–	–	Not performed	Died	2002 [12]
F	41	190/130	41.0	–	–	–	–	–	Survived	2006 [9]
F	26	160/110	–	4.5	–	–	–	Not performed	Died	2008 [6]
M	39	240/140	–	5.5	41.0	49.6	–	Emergency	Survived	2008 [6]
F	52	300/200	39.9	10	–	–	–	Not performed	Died	2010 [13]
F	52	162/104	35.8	–	103.5	53.9	53.1	–	Survived	2010 [5]
M	27	160/120	–	5	–	–	–	Not performed	Died	2012 [14]
M	70	210/146	40.1	5	1047.5	61.5	103.5	5	Survived	Present

*M* male, *F* female, *S/DBP* systolic/diastolic blood pressure, *BT* body temperature, *Ad* adrenalin, *NAd* noradrenalin, *Dopa* dopamine, *Ref* reference number

^a^24 h urine correction

^b^Operation day following admission

Our patient presented with hypertension and ileus at onset, while subsequent computed tomography performed to examine the ileus revealed a left adrenal tumor. On admission, the patient did not present with high fever but showed a slight disturbance of consciousness and extremely high blood pressure. Despite blood pressure control with α1-blocker treatment after hospitalization, his body temperature increased and consciousness worsened. Moreover, liver dysfunction and respiratory distress developed. Thus, his symptoms met the diagnostic criteria of PMC. On the basis of these findings, we opted for emergency surgery before confirmation of blood catecholamine levels and Methoxy-Isobutyl-Isonitrile (MIBI) scintigraphy. His condition improved dramatically after surgery. He was weaned from continuous hemodiafiltration and respiration, catecholamine administration, and intubation at 4 days postoperatively.

Our case provides further support that surgical resection is important for preventing mortality in patients with PMC. Nevertheless, the appropriate timing of surgery remains unclear. As shown in Table 1, surgery on the day following admission may not be associated with outcome [2, 10, 11]. In addition, other vital signs and clinicopathological features do not appear to be associated with outcome [2, 5, 6, 9–14]. Thus, in addition to the difficulty in diagnosis, the optimal treatment strategies for patients with PMC remain unclear.

The proliferation index evaluated by Ki-67 is commonly used to assess the cell-cycle. A number of case series have reported a PI measured by Ki-67 staining of less than 1 % in benign pheochromocytoma [15, 16] (Table 2). By contrast, our case of benign pheochromocytoma showed an extremely high level of cell proliferation potential, with a PI of 9.7 %. The relationships between cell proliferation and pathological characteristics including hormone secretion, tumor size, and symptoms in benign pheochromocytoma are not fully understood, although PI was reported to be significantly associated with capsule invasion of pheochromocytoma [24]. The relationship between the aggressiveness of PMC and cell proliferation is unknown, as this is the first report on cell proliferation in PMC. Speculatively, the high proliferative potential despite benign tumor status may be a characteristic of PMC that contributes to its aggressive characteristics and severe symptoms in patients.

**Table 2:** Mean proliferation indexes of benign and malignant adrenal pheochromocytoma

Benign									
N	23^b^	16	93	21	20	–	41	44	14
PI (%)	1	0.75	0.21	0.9	<1	–	0.8	<1	2.2
Malignant									
N	10	15	36	4	3^c^	43	5	11	7
PI (%)	5	13.9	3.0	4.3	<1	7.8	1.4	–	14.1
Cut-off^a^	>3	≥3	>2.5	–	–	>5	>2	>5	–
Year	1998	2000	2000	2001	2003	2004	2008	2010	2012
Reference	[17]	[18]	[19]	[20]	[15]	[21]	[22]	[16]	[23]

*PI* proliferation index

^a^Cut-off of PI (%) between benign and malignant pheochromocytoma

^b^Including extra-adrenal tumors

^c^Patients without metastasis at diagnosis

PI is also a useful marker for distinguishing benign from malignant pheochromocytoma [16–19]. We identified nine studies in PubMed describing a PI cut-off in adrenal malignant pheochromocytoma tissues; the suggested cut-offs for predicting malignant behavior are shown in Table 2. The PI in the present case was relatively higher than the mean PI in malignant pheochromocytoma, indicating malignant and metastatic potential. However, no recurrence or metastatic mass was detected at 2 years postoperatively. Nevertheless, follow-up with imaging and hormonal studies are required as a part of the pheochromocytoma can recur in various organ types [25].

## Conclusions

We report a case of rapidly progressing and life-threatening PMC. Although it is generally difficult to decide on the surgical strategy for patients with PMC, emergency surgery is important for achieving a favorable outcome. Therefore, urologists and clinicians should be aware of this rare life-threatening condition. In addition, we speculate that the aggressive characteristics of PMC may be due to a high proliferative potential. Additional studies examining the pathological characteristics of the disease at the molecular level are required to determine the biological characteristics of PMC and to develop strategies for diagnosis and treatment.

## Consent

Written informed consent was obtained from the patient for the publication of this Case Report and any accompanying images.

## Abbreviations

PMC: pheochromocytoma multisystem crisis
PI: proliferation index
MIBI: methoxy-Isobutyl-Isonitrile
